# Comparison of the efficacy of two doses of dexmedetomidine as an adjunct to levobupivacaine in infraclavicular brachial plexus block: prospective double-blinded randomized controlled trial

**DOI:** 10.1186/s12871-022-01858-4

**Published:** 2022-11-05

**Authors:** Huda F. Ghazaly, Ahmed Alsaied A. Aly, Zaher Zaki Zaher, Mahmoud M. Hassan, Ahmed Abdelreheem Mahmoud

**Affiliations:** Anesthetic Department, Faculty of Medicine, Aswan University, Aswan, Egypt

**Keywords:** Adjunct, Analgesia, Dexmedetomidine, Levobupivacaine, Ultrasound-guided infraclavicular block

## Abstract

**Background:**

This prospective, double-blind, randomized, controlled trial compared the efficacy of two dexmedetomidine doses (50 and 100-μg) combined with levobupivacaine on sensory block duration in infraclavicular brachial plexus block. We hypothesized that perineural dexmedetomidine would extend sensory block duration dose-dependently.

**Methods:**

The study included 60 patients aged 20 to 60 years of both sex with an ASA I/II undergoing forearm and hand surgery. The patients were randomly assigned into three equal groups (*n* = 20) for ultrasound-guided infraclavicular brachial plexus block. The L group received 35-mL 0.5% levobupivacaine plus normal saline, the LD50 group received 35-mL 0.5% levobupivacaine plus 50-μg dexmedetomidine, and the LD100 group received 35-mL 0.5% levobupivacaine plus 100-μg dexmedetomidine. Patients were investigated for onset and duration of sensory blockade, time to first postoperative rescue analgesia, and the total 24-h postoperative morphine requirement.

**Results:**

The LD100 group had a longer sensory block duration (15.55 ± 1.1 h; 95% confidence interval (CI), 15.04–16.06) than the LD50 group (12.8 ± 1.2 h; 95% CI, 12.24–13.36 h) (*p* < 0.001) or the L group (9.95 ± 1.05 h; 95% CI, 9.46–10.44 h) (*p* < 0.001). The LD100 group took longer to request postoperative rescue analgesia and required fewer postoperative morphine doses than the LD50 and L groups (*P* < 0.001).

**Conclusions:**

Sensory block duration was longer with perineural 100-μg dexmedetomidine as an adjunct to levobupivacaine than with 50-μg dexmedetomidine.

**Trial registration:**

This study was approved by the Ethics Committee of Aswan University Hospital (approval number: aswu/125/4/17) (date of registration: 04/04/2017). Furthermore, the trial was retrospectively registered at ClinicalTrial.gov (NCT04729868) with a verification date of January 2021.

## Background

Peripheral nerve blocks are widely used in upper limb surgery because they improve postoperative pain control and reduce the possibility of delirium or cognitive dysfunction [1]. The infraclavicular technique has the potential benefit of a compact anatomical distribution of plexus structures, allowing for a single injection of local anesthetics (LAs) and reducing the risk of pneumothorax [2].

On the other hand, because the length of the sensory block following a single injection of LAs is often insufficient to obviate the need for postoperative opioids, several adjuvants have been used to extend the duration of nerve blocks [3]. Dexmedetomidine is an alpha-2 adrenergic receptor agonist used as an adjuvant to LAs [4, 5]. The effectiveness of dexmedetomidine in developing the time of a brachial plexus block during upper limb surgery has been investigated in several studies. It was hypothesized that it has a synergistic effect with LAs and extends the duration of their activity [6–10]. However, the optimal dose of dexmedetomidine for brachial plexus blockade is a matter of debate.

A previous study that used different doses of dexmedetomidine as an adjuvant to levobupivacaine in supraclavicular brachial plexus block reported 60-µg dexmedetomidine improved block qualities clinically with minimal side effects [11]. Also, Esmaoglu et al. [12] have reported that adding 100-µg dexmedetomidine to levobupivacaine prolonged the duration of axillary brachial plexus blocks and speeded up the onset of sensory and motor blocks.

Notably, the effects of different dexmedetomidine doses with levobupivacaine in infraclavicular brachial plexus block have not been adequately investigated. This double-blind, randomized, controlled trial was designed to compare the efficacy of two dexmedetomidine doses (50 and 100-μg) with levobupivacaine in ultrasound-guided infraclavicular brachial plexus block and to determine the dose that provides a balance between improvement in block parameters, hemodynamic alterations, and sedation. The primary outcome in this study is the duration of sensory blockade. Moreover, secondary effects include sensory and motor blockade onset time, elapsed time to the first postoperative request for rescue analgesia, postoperative analgesic needs in the first 24 h, hemodynamic alterations, and sedation.

## Methods

### Ethics and registration

This study was approved by the Ethics Committee of Aswan University Hospital (approval number: aswu/125/4/17) (date of registration: 04/04/2017). Furthermore, this trial was retrospectively registered at ClinicalTrial.gov (NCT04729868) with a verification date of January 2021. Participants were aware of the study's objectives, risks, and benefits before signing a written informed consent form. All methods were performed according to the guidelines of the Declaration of Helsinki and its later amendments.

### Patient inclusion and exclusion criteria

Patients aged 20–60 years of both sex who had an American Society of Anesthesiologists (ASA) physical status of I/II and scheduled for forearm and hand surgery were recruited. Patients with a history of brachial plexus injury, coagulopathy, allergy to the study drugs, hepatic or renal insufficiency, respiratory or cardiac disorders, seizures, pregnancy, or local infections at the site where the block needle was to be inserted were excluded from the study.

### Randomization

Randomization was established using computer-generated randomization tables to allocate patients into one of three equal groups, and the group allocation was concealed in sealed opaque envelopes. Patients were allocated into one of three equal groups: the L group consisted of patients who received anesthesia with 35-mL 0.5% levobupivacaine plus 1 mL normal saline; the LD50 group included patients who received anesthesia with 35-mL 0.5% levobupivacaine plus 50-µg dexmedetomidine, and the LD100 group included patients who received anesthesia with 35-mL 0.5% levobupivacaine plus 100-µg dexmedetomidine.

An investigator who was not involved in either the block performance or the outcome evaluation received serially numbered sealed envelopes labeled L, LD50, or LD100 for preparing the anesthetic mixture to be administered. The total volume of dexmedetomidine was adjusted to 1 ml to ensure that the same volume of the anesthetic mixture was used for all groups. The patients, operator performing the block, and data collectors were blinded to the group assignment.

### Anesthesia

The procedure was fully explained to the patients primarily for ethical aspects and to ensure cooperation and acceptance of being awake during surgery. Moreover, the visual analog scale (VAS) was explained (the VAS is a straight, vertical 10-cm line with the bottom point representing “no pain” (0 cm) and the top point representing “the worst pain you could ever have” (10 cm). Before the procedure, for a minimum of 6 h, all patients were given nothing by mouth. Standard monitoring using an electrocardiogram, noninvasive blood pressure, and pulse oximetry (SpO2) was connected to the patients upon arrival at the operating room, and the displayed data were recorded before surgery. A 20-G cannula was inserted into the contralateral hand, and Ringer’s lactate was infused. Patients were sedated using 0.05-mg/kg intravenous midazolam hydrochloride and fentanyl in doses ranged from 0.5 to 1 μg/kg based on the patient response. During surgery, a nasal catheter with a 3-L/min oxygen supply was used.

The ultrasound-guided infraclavicular brachial plexus block was performed by a single trained author who was blinded to the nature of the anesthetic mixture. While the patient was supine and the arm was abducted to 90°, the deltopectoral region was scanned using an ultrasound machine (SonoScape, model A5, China) equipped with a high-frequency (> 10 MHz) linear probe. Then, a 20-G, 120-mm, non-cutting-tip echogenic needle (SonoPlex STIM, Germany) was introduced and advanced in-plane until imaged.

The needle was first aimed at the medial cord, which runs between the axillary artery and vein, and numbed using the injected LAs (12 ml). Then, the needle was guided to the lateral cord before injecting the LA solution (12 ml). It was then advanced to the posterior cord, and more LAs were deposited (12 ml). During injections, the operator sonographically tracked LAs around the cords and always kept the needle's image in view. The following anesthetic mixtures were administered according to the patient’s study group assignment: 35-mL 0.5% levobupivacaine plus 1 mL normal saline (control group), 50-μg or 100-μg dexmedetomidine (study groups).

### Outcome measures

The primary endpoint was the duration of sensory blockade. In contrast, the secondary ones included the duration of motor blockade, sensory and motor blockade onset time, elapsed time to first postoperative request for rescue analgesia, postoperative analgesic needs in the first 24 h, hemodynamic monitoring, and sedation scores. All patients were evaluated for the following conditions:

• Sensory block duration (h) was the time elapsed between the onset of the sensory block and the restoration of sensation at the surgical site, while the time elapsed between the onset of the motor block and the restoration of global mobility in hand and wrist was measured as the duration of the motor block (h).
• Onset time of sensory blockade was measured by loss of sensation to a gauze soaked in normal iced saline using a 3-point scale: 0 = complete loss of sensation, 1 = partial loss of sensation, and 2 = normal sensation, along the middle of the dermatomal distribution of the musculocutaneous, median, radial, and ulnar nerves, and the onset time of motor blockade was measured using the modified Bromage scale (0 = normal motor function, 1 = reduced motor strength but able to move fingers, 2 = complete motor block) [13–15].
• Heart rate (beat/min), oxygen saturation, and mean arterial blood pressure (mmHg) were measured before the block; 5, 10, 20, 30, 40, 50, and 60 min after the block; and 6, 12, and 24 h later.
• VAS was used to rate pain control at the 1st, second, fourth, sixth, 12th, and 24th hours after surgery. When the VAS reached 3 cm, rescue analgesia in the form of 0.05-mg/kg morphine sulfate was administered intravenously; time to the first postoperative request for rescue analgesia and the total morphine consumption in the first 24 h were recorded.
• The Modified Ramsay Sedation Scale (m-RSS) was used to assess the sedation score every hour until four hours after the block [16].
• The patients were examined for any complications that could arise during or after the procedure. Bradycardia was defined as a heart rate of fewer than 50 beats per minute and was treated with a 0.5–1-mg intravenous bolus of atropine. Hypotension was described as a 30% decrease in the mean arterial pressure (MAP) from baseline or MAP less than 60 mmHg and was treated with 6-mg intravenous ephedrine in increments as needed.

### Sample size and statistical analysis

Based on a pilot study involving 15 patients, the sample size was calculated using G*Power 3 software. The primary outcome measure in this study was the duration of sensory blocks. The minimum sample size for a one-tailed test with a power of 80% and a type I error of 5% (α = 0.05 and β = 80%) was 54 people divided into three equal groups. Each group required 18 patients to detect a 0.5 effect size in the mean duration of the sensory block (8, 12, and 14 h). We added 10% of the total study population to compensate for dropouts.

Data were analyzed using Statistical Package for the Social Sciences (version 26; IBM Corp., Armonk, NY, USA). Normality tests (the Kolmogorov–Smirnov, and Shapiro–Wilk tests) were performed, and data (i.e., age, ASA physical status, duration of surgery, duration of sensory blockade, the onset of sensory blockade, duration of motor blockade, the onset of motor blockade, time to the first request for postoperative rescue analgesia, total postoperative morphine sulfate needs, mean arterial blood pressure, mean heart rate, and arterial oxygen saturation) were normally distributed. In contrast, data on VAS were not normally distributed. Nominal data were expressed as percentages; differences between all groups under study were detected using the chi-square test. Continuous data were expressed as mean ± standard deviation or median (range). Differences between all groups under study were detected using one-way ANOVA; multiple comparisons between every two groups were detected using the least significant difference (LSD) post hoc multiple comparisons for parametric data. In contrast, differences between all groups under study were detected using the Kruskal–Walls test; multiple comparisons between every two groups were detected using the Mann–Whitney test for nonparametric data. Spearman’s correlation coefficients were used to analyze the correlation between different parameters within the groups under study. Kaplan–Meier curve was used to estimate the median survival time. The Log-rank test was used to compare survival curves between the categories of the explanatory variables. *P*-values of less than 0.05 were used to denote statistical significance.

## Results

**Fig. 1:**
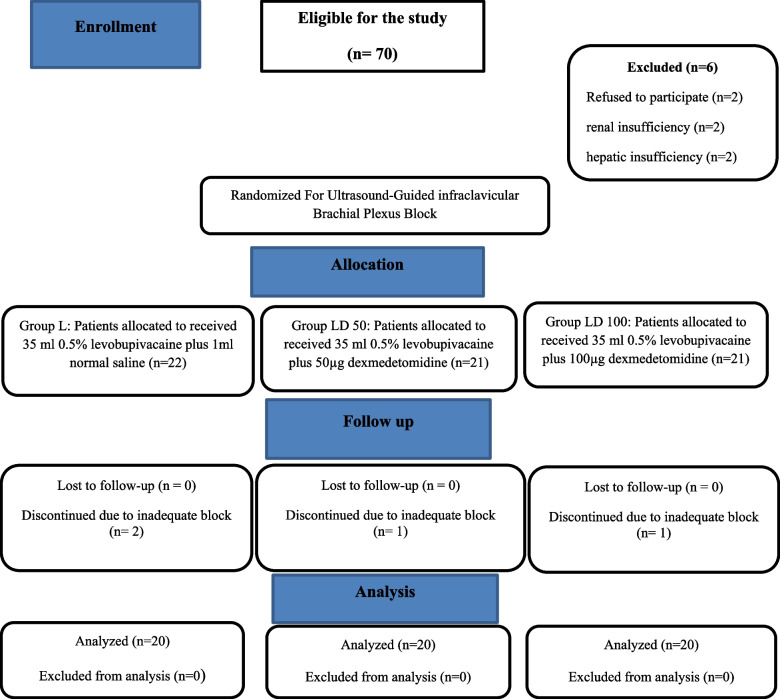
CONSORT flow chart displays the number of patients at each study stage

Seventy patients were eligible for this study. The authors ruled out sex patients from the study: two refused to participate and four did not meet the eligibility criteria (two had renal and two had hepatic insufficiency). Finally, 64 patients were enrolled in this study and randomly divided into three equal groups for ultrasound-guided infraclavicular brachial plexus block using 35-mL 0.5% levobupivacaine plus 1-mL normal saline (control group), 50-μg or 100-μg dexmedetomidine (study groups). Four patients were withdrawn from the trial (two from the L group, one from the LD50 group, and one from the LD100 group) due to an inadequate block and the need for general anesthesia after surgical incision, leaving 60 patients to complete the study (Fig. 1). Basic and surgical characteristics of patients were comparable between the three study groups (Table 1).

**Table 1:** Data on the patients and surgical characteristics

	Group						
	L (*N* = 20)		LD50 (*N* = 20)		LD100 (*N* = 20)		*P*-value
Gender	No	%	No	%	No	%	
Male	11	55.0	14	70.0	14	70.0	0.517
Female	9	45.0	6	30.0	6	30.0	
Age(years)							
Mean (SD)	36.15(10.47)		35.8 (11.44)		37.75 (11.35)		0.839
ASA classification	No	%	No	%	No	%	0.918
ASA grade 1	15	75.0	14	70.0	15	75.0	
ASA grade 2	5	25.0	6	30.0	5	25.0	
Weight (kg)							
Mean (SD)	85 (4.54)		88.75 (5.37)		88.1(7.2)		0.102
Duration of surgery (h)							
Mean (SD)	2.2 (0.83)		1.9 (0.79)		2.15(0.75)		0.442

Continuous variables are presented as mean (SD). Categorical variables are presented as numbers (%). L group, 0.5% levobupivacaine plus 1-mL normal saline; LD50 group, 0.5% levobupivacaine plus 50-µg dexmedetomidine; LD100 group, 0.5% levobupivacaine plus 100-µg dexmedetomidine; ASA, American Society of Anesthesiologists; SD, standard deviation

### Infraclavicular brachial plexus block characteristics:The primary endpoint

**Fig. 2:**
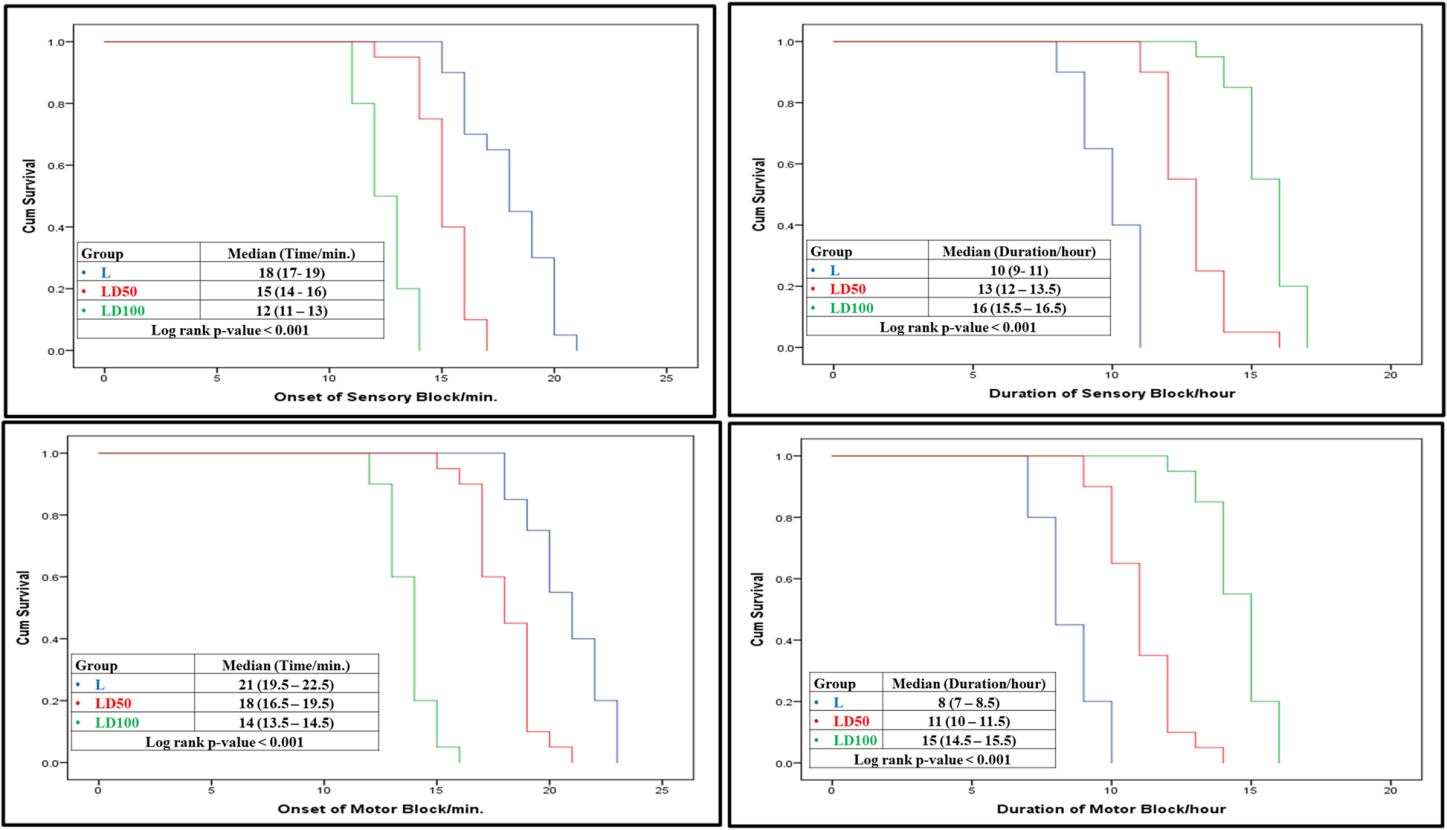
Kaplan Mayer Curve for the effect of different dexmedetomidine doses on the onset and duration of both sensory and motor blockade

the mean duration of sensory block, was significantly longer in the LD100 group (15.6 ± 1.1 h; 95% confidence interval (CI), 15.04–16.06 h) than the LD50 (12.8 ± 1.2 h; 95% CI, 12.24–13.36) (LD100 vs LD50, *P*-value < 0.001)and L (9.95 ± 1.05 h; 95% CI, 9.46–10.44 h) (LD100 vs L, *P*-value < 0.001) groups, and it was longer in the LD50 group than that in the L group (LD50 vs L, *P*-value < 0.001). The mean sensory block onset time was significantly faster in the LD100 group (12.5 ± 1.05 min; 95% CI, 12.01–12.99 min) than the LD50 (15.15 ± 1.18 min; 95% CI, 14.6–15.7 min) (LD100 vs LD50, *P*-value < 0.001) and L (18.05 ± 1.88 min; 95% CI, 17.17–18.93 min) (LD100 vs L < 0.001, *P*-value < 0.001) groups, and it was faster in the LD50 group than the L group (LD50 vs L, *P*-value < 0.001) (Table 2 and Fig. 2).

**Table 2:** Primary and secondary outcomes

Data	Group		
	L (*N* = 20)	LD50 (*N* = 20)	LD100 (*N* = 20)
Duration of sensory blockade (h) [95% CI]	9.95 ± 1.05 [9.46–10.44]	12.8 ± 1.2* [12.24–13.36]	15.55 ± 1.1*† [15.04–16.06]
The onset of sensory blockade(min) [95% CI]	18.05 ± 1.88 [17.17–18.93]	15.15 ± 1.18* [14.6–15.7]	12.5 ± 1.05*† [12.01–12.99]
Duration of motor blockade(h) [95% CI]	8.45 ± 1.05 [7.96–8.94]	11.05 ± 1.28* [10.45–11.65]	14.55 ± 1.1*† [14.04–15.06]
The onset of motor blockade(min) [95% CI]	20.75 ± 1.74 [19.93–21.57]	18.05 ± 1.43* [17.38–18.72]	13.75 ± 1.02*† [13.27–14.23]
Time to the first request for postoperative rescue analgesia (h)	6.27 ± 0.88	6.86 ± 0.94	12.18 ± 1.4*†
Total postoperative morphine sulfate needs (mg)	12.7 ± 0.92	9.9 ± 0.79*	5.8 ± 0.77*†
m-RSS 1st hour	2 ± 0	2 ± 0	3 ± 0*†
m-RSS2nd hour	2 ± 0	2 ± 0	3 ± 0*†
m-RSS 3^rd^ hour	2 ± 0	2 ± 0	2.95 ± 0.22*†
m-RSS 4thhour	2 ± 0	2 ± 0	2.85 ± 0.37*†

Continuous variables are presented as mean ± SD. L group: 0.5% levobupivacaine plus 1 ml normal saline; LD50 group: 0.5% levobupivacaine plus 50 µg dexmedetomidine; LD100 group: 0.5% levobupivacaine plus 100 µg dexmedetomidine; *Statistical significance compared with the L group. †Statistical significance compared with L50 group

likewise, the LD100 group had a faster onset of motor block (13.75 ± 1.02 min; 95% CI, 13.27–14.23 min) than the LD50 (18.05 ± 1.43 min; 95% CI, 17.38–18.72 min) (LD100 vs LD50, *P*-value < 0.001) and L (20.75 ± 1.74 min; 95% CI, 19.93–21.57 min) (LD100 vs L, *P*-value < 0.001) groups. The LD100 group had a longer motor block (14.55 ± 1.1 h; 95% CI, 14.04–15.06 h) than the LD50 (11.05 ± 1.28 h, 95% CI, 10.45–11.65 h) (LD100 vs LD50, *P*-value < 0.001) and L (8.45 ± 1.05 h; 95% CI, 7.96–8.94 h) (LD100 vs L, *P*-value < 0.001) groups, whereas the LD50 group had a longer motor block than the L group (LD50 vs L, *P*-value < 0.001) (Table 2 and Fig. 2).

Furthermore, there was a strong positive correlation (*p* < 0.001) between sensory (*r* = 0.912) and motor (*r* = 0.909) blockade durations and dexmedetomidine doses, indicating that the sensory and motor blockade durations increased when dexmedetomidine doses were increased. On the other hand, a significant negative correlation (*p* < 0.001) was observed between sensory (*r* =  − 0.873) and motor (*r* =  − 0.885) blockade onset times, indicating that as dexmedetomidine doses were increased, the sensory and motor blockade onset times decreased.

### Postoperative VAS score, time to the first request for postoperative rescue analgesia, and total postoperative morphine sulfate need in 24 h

**Fig. 3:**
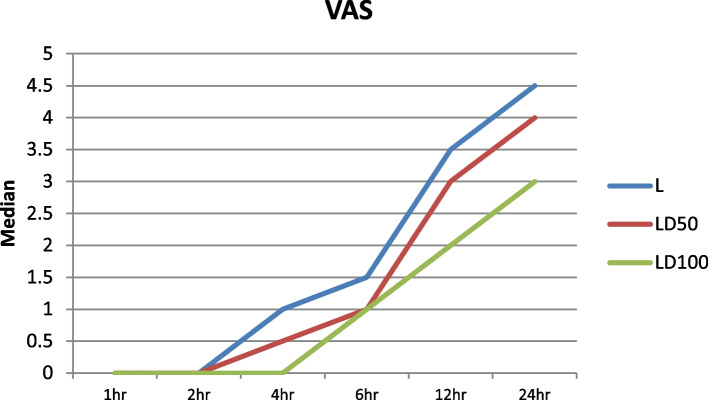
Postoperative VAS scores for the study groups. L group (patients received 0.5% levobupivacaine plus 1-mL normal saline); LD50 group (patients received 0.5% levobupivacaine plus 50-µg dexmedetomidine); LD100 group (patients received 0.5% levobupivacaine plus 100-µg dexmedetomidine). The postoperative VAS score in the LD100 group was lower than that in the L group at 4 (*p* < 0.005), 6 (*p* < 0.001), 12 (*p* < 0.003), and 24 (*p* < 0.001) hours after surgery and was significantly lower than that in the LD50 group only at the 24th (*p* < 0.042) hour

The postoperative VAS score in the LD100 group was significantly lower than that in the L group at the 4th, 6th, 12th, and 24th hours after surgery (*P* = 0.005, *P* = 0.005, *P* = 0.003, and *P* = 0.001, respectively); and lower than LD50 group at the 24 h (*P* = 0.042) (Fig. 3). Its clinical significance, however, is limited because all patients were given a multimodal analgesic regimen supplemented by intravenous rescue analgesia to maintain a VAS score ≤ 3. The mean time to the first request for postoperative rescue analgesia was longer in the LD100 group than in the LD50 (LD100 vs LD50, *P*-value < 0.001) and L (LD100 vs L, *P*-value < 0.001) groups. Postoperative morphine requirement was also lower in the LD100 group than the LD50 (LD100 vs LD50, *P*-value < 0.001) and L (LD100 vs L, *P*-value < 0.001) groups, with the L50 group requiring less postoperative morphine than the L group (LD50 vs L, *P*-value < 0.001) (Table 2).

### Sedation

There was a significant difference in m-RSS scores between study groups until the fourth hour after surgery; patients in the LD100 group were more sedated than those in the LD50 (LD100 vs LD50, *P*-value < 0.001) and L (LD100 vs L, *P*-value < 0.001) groups (Table 2).

### Hemodynamic findings

From 10 min to 2 h after infraclavicular brachial plexus block, the mean arterial blood pressure in the LD100 group was lower than that in the L group; however, it was lower than that in the LD50 group only 20, 40, and 50 min after infraclavicular brachial plexus block. None of the patients required pharmaceutical treatment. The mean heart rate in the LD100 group was significantly lower than that in the LD50 and L groups from 10 min to 2 h after infraclavicular brachial plexus block. The lowest heart rate was 60.8 ± 8.33 beats/min 30 min after the block, which did not require any treatment. The arterial oxygen saturation was comparable between the study groups, and no desaturation episodes (SpO2 < 90%) were observed.

### Adverse events

postoperative nausea or vomiting occurred in 10% of patients in the LD100 group and only 5% in the L and LD50 groups.

## Discussion

In the context of an ultrasound-guided infraclavicular brachial plexus block with various doses of dexmedetomidine with levobupivacaine, the current study showed that patients in the dexmedetomidine groups had a longer duration of sensory and motor blockade and faster onset in a dose-dependent manner than those in the levobupivacaine group.

Higher dexmedetomidine doses (100 µg) had a significant positive correlation with the duration of sensory and motor blockade and a significant negative correlation with the onset of sensory and motor blockade. The time to first request postoperative rescue analgesia was longer in the LD100 group, and the required postoperative morphine doses were smaller than those in the LD50 and L groups. Furthermore, the LD100 group had lower mean arterial blood pressure and heart rate and was more sedated than the other groups, and no patients required pharmacological intervention.

Our results agreed with those of Balakrishnan et al. [11], who conducted a study on 120 patients divided into four groups and administered plain levobupivacaine and 30-μg, 60-μg and 100-μg dexmedetomidine along with levobupivacaine. They found that the 100-μg dexmedetomidine group had a statistically significant increase in sensory and motor blockade durations, a decrease in onset time, and a prolongation of analgesia duration compared with the other three groups. Reddy et al. [17] also evaluated two doses of dexmedetomidine, 50 μg and 100 μg, added to 0.5% levobupivacaine, on 120 patients undergoing upper limb surgeries under supraclavicular brachial plexus block. They reported that adding 100-μg dexmedetomidine to 0.5% levobupivacaine lengthened the duration of sensory and motor blocks and accelerated their onset. Rescue analgesia in the form of diclofenac sodium injection was required in 20 patients (33.33%) in the 50-μg group and nine patients (15%) in the 100-μg group.

Furthermore, a recent meta-analysis that included 18 randomized controlled trials (*n*= 1,014) conformed to the current findings on adding dexmedetomidine (50–100 μg) to LAs in brachial plexus block. They have found that in patients who received 100-μg dexmedetomidine, the mean sensory block duration increased by 257 min, the mean motor block duration increased by 242 min, and the mean time to the first demand for analgesia increased by 266 min [18]. A comparable meta-analysis by Hussain et al. [19] on 18 studies (*n* = 1,092) found that the addition of dexmedetomidine to LAs increased the duration of sensory (261.41 min) and motor (200.9 min) blocks, reduced the onset of sensory (3.19 min) and motor (2.92 min) blocks, increased the duration of analgesia (289.31 min), and significantly reduced postoperative analgesic requirement 24 h after the block compared with the control; however, three studies have found no significant difference between the dexmedetomidine and control groups.

Likewise, Zhang et al. [7] found a prolonged duration of analgesia in patients who received a higher dose of dexmedetomidine (100 μg) in 40-mL 0.33% ropivacaine than in patients who received 50-μg dexmedetomidine in axillary brachial plexus block. According to Keplinger et al., [8] 50-, 100-, and 150-μg dexmedetomidine increased the duration of sensory block by 60%, 72%, and 57%, respectively, compared with ropivacaine alone (*p* < 0.05). Moreover, Abdulatif et al. [20] concluded that adding 50- and 75-μg dexmedetomidine was associated with an increase in the duration of sensory and motor blocks, a decrease in the time to the onset of sensory and motor blocks, an increase in the time to the first request of morphine, and a decrease in postoperative morphine consumption. The total postoperative morphine requirement was lower in the 75-μg and 50-μg groups than in the control group. However, Aksu R et al. [21] anesthetized 50 patients with a supraclavicular block using 30-mL plain bupivacaine versus bupivacaine plus dexmedetomidine and found no difference in the onset of sensory or motor block, duration of analgesia, or duration of sensory or motor block. This is most likely because dexmedetomidine group received 15-mL 0.33% bupivacaine and 1-μg/kg dexmedetomidine vs. control group, received 30-mL 0.33% bupivacaine.

Regarding hemodynamic findings, it was consistent with a recent meta-analysis [22], which reported that perineural dexmedetomidine increased the risk of bradycardia and hypotension, both of which were transient and could be reversed using atropine or ephedrine. This may be due to dexmedetomidine inhibiting sympathetic outflow and norepinephrine release via alpha-2 subtype receptors. Aksu et al. [21] have found a statistically significant decrease in heart rates after supraclavicular brachial plexus block in the group who received 15-mL 0.33% bupivacaine and 1-µg/kg dexmedetomidine compared with those in the group who received 30-mL 0.33% bupivacaine 5, 15, 30, 45, 60, and 90 min after block and a statistically significant decrease in MAP in the bupivacaine dexmedetomidine group 15 min after block only. Furthermore, considerable bradycardia has been reported when using perineural 0.5% levobupivacaine plus 100-μg dexmedetomidine, which agrees with our findings [17].

In a meta-analysis, Hussain et al. [19] reported that when dexmedetomidine was greater than 50 μg, more cases had intraoperative bradycardia; however, dexmedetomidine doses of 50 μg or less failed to show significance in cases of bradycardia when compared with the control group. The occurrence of intraoperative hypotension after the addition of dexmedetomidine was insignificant in comparison with the control group, regardless of dosage (50 μg or more than 50 μg).

The study found that patients in the LD100 group were more sedated in a dose-dependent manner than those in the LD50 and L groups. A study by Reddy found that the LD100 group experienced significantly more sedation than the LD50 group [17]. Furthermore, Balakrishnan et al. [11] found a significant increase in sedation scores in the LD100 group compared with those in other groups. However, Abdulatif et al. found no statistically significant differences in sedation among the four study groups (using 25-mL bupivacaine combined with normal saline in the control group and 25-μg, 50-μg, and 75-μg dexmedetomidine in three treatment groups, respectively). This may be due to the use of relatively low doses of dexmedetomidine. [20]

This study has encountered several limitations. First, we cannot conclude the effects of dexmedetomidine on patients with renal, hepatic, or cardiac impairment based on our findings. Second, monitoring the bi-spectral index (BIS) values would have provided a more objective sedation state evaluation than the mRSS [23]. Third, even though patients received perioperative sedation in addition to the additional effect of dexmedetomidine, we did not use intraoperative end-tidal CO2 monitoring. Finally, we did not measure the plasma levels of the study drugs.

## Conclusions

Based on the findings of this study, perineural infiltration of 50-μg and 100-μg dexmedetomidine as an adjunct to levobupivacaine increases the sensory and motor block duration. It reduces the onset time in a dose-dependent manner. We conclude that 100-μg dexmedetomidine has a longer sensory block duration than 50-μg dexmedetomidine, significantly extending analgesia duration and reducing the need for additional analgesics.

## Data Availability

All data used and analyzed in this study are available from the corresponding authors upon reasonable request.

## Abbreviations

ASA: American Society of Anesthesiologists
mRSS: Modified Ramsay Sedation Scale
VAS: Visual analog scale
